# Sleep enhances inhibitory behavioral control in discrimination learning in rats

**DOI:** 10.1007/s00221-013-3797-5

**Published:** 2013-12-10

**Authors:** Margarita Borquez, Jan Born, Victor Navarro, Ronald Betancourt, Marion Inostroza

**Affiliations:** 1 Departamento de Psicología, Universidad de Chile, Santiago, Chile; 2 Center for Integrative Neuroscience, Institute of Medical Psychology and Behavioral Neurobiology, University of Tübingen, Otfried-Müller-Strasse 25, 72076 Tübingen, Germany

**Keywords:** Inhibitory control, Discrimination learning, Sleep, Rats

## Abstract

Sleep supports the consolidation of memory, and it has been proposed that this enhancing effect of sleep pertains in particular to memories which are encoded under control of prefrontal–hippocampal circuitry into an episodic memory system. Furthermore, repeated reactivation and transformation of such memories during sleep are thought to promote the de-contextualization of these memories. Here, we aimed to establish a behavioral model for the study of such sleep-dependent system consolidation in rats, using a go/nogo conditional discrimination learning task known to essentially depend on prefrontal–hippocampal function. Different groups of rats were trained to criterion on this task and, then, subjected to 80-min retention intervals filled with spontaneous morning sleep, sleep deprivation, or spontaneous evening wakefulness. In a subsequent test phase, the speed of relearning of the discrimination task was examined as indicator of memory, whereby rats were either tested in the same context as during training or in a different context. Sleep promoted relearning of the conditional discrimination task, and this effect was similar for testing memory in the same or different context (*p* < 0.001). Independent of sleep and wakefulness during the retention interval, animals showed faster relearning when tested in the same context as during learning, compared with testing in a different context (*p* < 0.001). The benefitting effect of sleep on discrimination learning was primarily due to an enhancing effect on response suppression during the nogo stimulus. We infer from these results that sleep enhances memory for inhibitory behavioral control in a generalized context-independent manner and thereby might eventually also contribute to the abstraction of schema-like representations.

## Introduction

Sleep plays a pivotal role for the consolidation of memory (Stickgold 2005; Diekelmann and Born 2010). The benefitting effect of sleep appears to be selective, enhancing in particular memory involving prefrontal–hippocampal circuitry at encoding, i.e., memories for episodes (Inostroza and Born 2013). Episodic memory basically refers to the recall of events as they were uniquely experienced in a specific spatiotemporal context (Tulving 1983). It has been proposed that sleep promotes the maintenance of such memories by an active system consolidation process originating from the neuronal reactivation of representations newly encoded in the hippocampus which serves the binding of events into spatiotemporal context (Wilson and McNaughton 1994; Rasch et al. 2007; Lewis and Durrant 2011; Inostroza and Born 2013). The reactivations are assumed to produce two different effects: They initially strengthen the hippocampal representation, and they more gradually promote redistribution toward preferential representation of the memory in extra-hippocampal mainly neocortical networks. The redistribution is thought to be accompanied by a transformation of the episodic memories, leading to the generation of schema-like representations that are less dependent on specific contexts (Nadel et al. 2000; Winocur et al. 2010). Although overall convergent evidence exists that sleep supports the redistribution of memory representations (e.g., Gais et al. 2007; Takashima et al. 2006; Rasch and Born 2013), it is less well studied whether sleep concurrently supports the de-contextualization of memory. To our knowledge, there are so far only two human studies that provide preliminary evidence in support of this assertion (Cairney et al. 2011; Deliens et al. 2013).

In the present study, we sought to examine the effect of sleep on the consolidation and presumed de-contextualization of memory in a rat model. As memory task, we adopted a conditional discrimination paradigm in which the subject learns to respond in the presence of a discriminative stimulus and to inhibit the response in the absence of this stimulus (Skinner 1969). Acquisition of such tasks involving go/nogo discriminative learning has been shown to involve prefrontal–hippocampal circuitry, whereby in particular learning of response, inhibition requires prefrontal executive control (Bari and Robbins 2013; Chudasama et al. 2012, Munakata et al. 2011; Dalley et al. 2004). Indeed, inhibitory response control represents an executive function widely implemented to regulate emotion and goal-directed behavior in very different contexts (Bari and Robbins 2013; Herry et al. 2010), and it might be such involvement of executive control in a learning task that especially favors processes of generalization and de-contextualization to occur during the post-encoding consolidation of the task. Prefrontal neuron assemblies active during inhibitory control in a rule shift task have been demonstrated to be reactivated during subsequent sleep, in conjunction with the occurrence of hippocampal sharp wave–ripples (Benchenane et al. 2010; Peyrache et al. 2009). However, those studies did not examine whether learning on these tasks is actually improved by sleep.

Here, using a conditional discrimination paradigm, we aimed at clarifying whether sleep compared with a post-training wake interval promotes memory formation in a task requiring inhibitory control learning and whether sleep also supports the generalization of learning to a different context. The experiments served as a first step to establish a behavioral model for the study of system consolidation during sleep in rats.

## Materials and methods

### Animals

Subjects were 50 experimentally naïve, male Sprague–Dawley rats, approximately 3-month old and with a mean weight of 275 g. The rats were obtained from the breeding colony at the Facultad de Ciencias Biomédicas of the Pontificia Universidad Católica de Chile, and kept in individual cages. Throughout the entire experiment, rats were food-deprived to 85 % of their free-feeding weights, but water was freely available. The rats were kept in a controlled 12-h light/12-h dark cycle with lights switched on at 07:00 a.m. All experimental procedures were approved by the ethics committee of the Universidad de Chile.

### General procedure

**Fig. 1:**
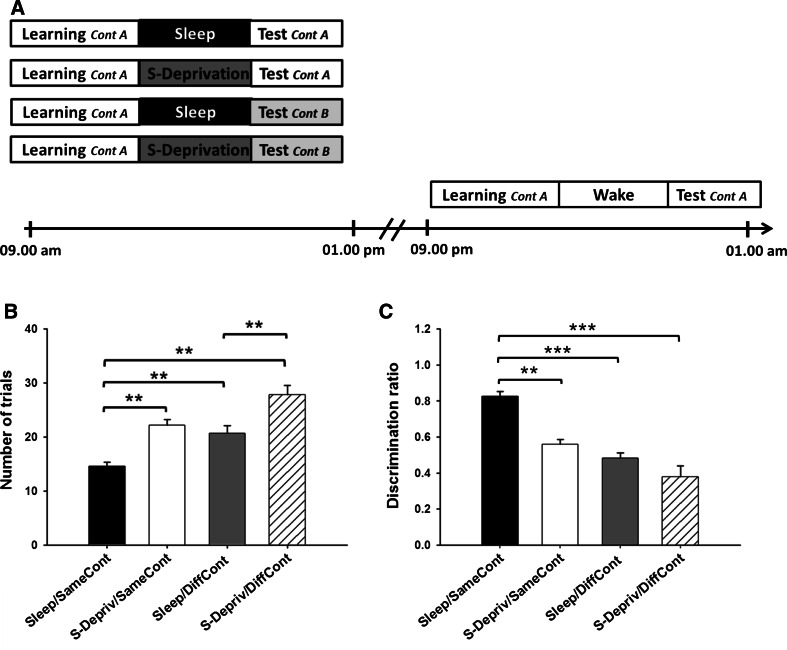
**a** Experimental design (see text for details). Five different groups of rats were tested in different conditions. Generally, testing comprised a learning phase in which the rats were trained on the go/nogo conditional discrimination task, followed by an 80-min retention period in which the rats slept (Sleep) or were awake (S-Deprivation, Wake), and a final test phase, in which memory for the conditional discrimination task was tested by evaluating relearning performance. Testing after sleep and sleep deprivation took place either in the same context as during learning (Cont A) or in a different context (Cont B). **b** Mean (±SEM) number of trials needed to reach the learning criterion and **c** discrimination ratios on the conditional discrimination task during the test phase separately for the four groups of rats. Note fastest relearning and highest discrimination ratios for the Sleep/SameCont group in comparison with all other groups. ***p < 0.001; **p < 0.01; *p < 0.05, for pairwise comparisons between groups

The experimental session comprised three phases: (1) a learning phase in which the training on the go/nogo conditional discrimination was performed; (2) an 80-min retention interval in which the rats slept (Sleep) or were awake (S-Deprivation, Wake); and (3) a final test phase in which the memory for the conditional discrimination task was tested by evaluating relearning performance (Fig. 1a provides a summary of the experimental design and procedures). The animals were randomly allocated to five different groups: the Sleep/Same Context group (Sleep/SameCont, *n* = 11), the Sleep-Deprivation/Same Context group (S-Depriv/SameCont, *n* = 10), the Wake/Same Context group (Wake/SameCont, *n* = 10), the Sleep/Different Context group (Sleep/DiffCont, *n* = 10), and the Sleep-Deprivation/Different Context group (S-Depriv/DiffCont, *n* = 10). Rats of all groups were first trained to a criterion on the go/nogo conditional discrimination task (learning phase). The learning phase took place between 09:00 a.m. and 01:00 p.m. of the light phase, i.e., during the natural rest phase of the rats, for all groups, except for the rats of the Wake/SameCont group which were trained between 09:00 p.m. and 01:00 a.m. of the dark cycle, i.e., during the rats’ natural activity phase. The Wake/SameCont group was introduced to control for possible confounding influences of the circadian rhythm.

The learning phase was followed by an 80-min retention interval during which the rats of the Sleep/SameCont and Sleep/DiffCont groups were allowed to sleep in their home cages, whereas rats of the in S-Depriv/SameCont and S-Depriv/DiffCont groups were sleep deprived during this time. The rats of the Wake/SameCont group were spontaneously awake during this interval. Sleep deprivation was achieved by a “gentle handling” procedure to avoid stress (Hagewoud et al. 2010). The procedure was initiated as soon as the animal showed signs of sleep and involved tapping on the cage, gently shaking the cage, or if necessary disturbing the sleeping nest. To assess sleep, animals were videotaped during the retention interval.

The retention interval was followed by the test phase where the rats’ performance on the go/nogo conditional discrimination task was re-evaluated. The Sleep/SameCont, S-Depriv/SameCont, and Wake/SameCont groups were tested during the test phase in the same context as during the learning phase, whereas the Sleep/DiffCont and S-Depriv/DiffCon groups were tested in a context different from that of the learning phase.

### Behavioral procedures and go/nogo conditional discrimination learning

On the day before the conditional discrimination training of the learning phase, during a 40-min session, each rat was trained in the operant chamber to eat the food pellets at the feeder and to perform a lever response through a shaping procedure, receiving a mean of altogether 90 food pellets. The light of the operant chamber was switched on during the whole procedure. First, food pellets were given independently of the rat’s behavior at a variable interval schedule with a mean interval of 60 s. Then, the lever press response was shaped by giving continuous reinforcement to successive approximations for this response until it was consistently executed. Later on this day, rats of the Sleep/DiffCont and S-Depriv/DiffCont groups were exposed for 20 min to the different experimental context (context B), to familiarize rats with this context which should prevent possible confounding effects of novelty experienced during the test phase.

In the go/nogo conditional discrimination task trained in the learning phase, a lever press response was reinforced with a food pellet only when the light of the operant chamber was switched on (discriminative Go stimulus; S1), but not when the light was off (delta Nogo stimulus; S2). The training comprised a mean of 34.3 trials, whereby a trial was defined by the presentation of one S1 followed by one S2. The average duration for S1 was (mean ± SEM) 35.4 ± 1.25 s and for S2 55.6 ± 1.32 s. The S2 duration, at the end, included an additional 5-sec interval which was introduced whenever the rat had (falsely) responded with a lever press and which should prevent that this false response became accidentally associated with the succeeding onset of S1. These added 5-sec intervals on average lengthened the duration of S2 when compared with S1. Reinforcement was given in a Fixed Rate 1 schedule (FR1, i.e., each lever press response was reinforced by one pellet) until the 4th trial and from then on was switched to a Variable Rate 4 schedule (VR4, i.e., every fourth lever press response was reinforced by one pellet) until the end of the training. Training was finished when the rat achieved the learning criterion, i.e., it did not make a lever press response during 10 consecutive S2 s.

### Test phase

During the test phase after the retention interval, the rats were exposed to the same conditional discrimination task as during the learning phase and relearning of the task was assessed, i.e., this test phase ended when the rat achieved the learning criterion of 10 consecutive trials in which no response to an S2 was made. The target-dependent variable was the number of trails the rat needed to achieve the criterion.

### Apparatus

The conditional discrimination task was trained in a standard operant chamber (Context A, Lafayette Instruments Co; 32.7 cm by 27.5 cm; height: 23.8 cm). Two of the 4 side walls were constructed in polycarbonate, whereas the other two were constructed in aluminum. The floor was made of 19 aluminum bars spaced apart by 1.5 cm. One aluminum wall had a stainless steel food cup centered inside a wider compartment. At the right side of this food cup compartment, an incandescent light (6 W, diameter 2.8 cm) was inserted in the wall 13 cm above the floor which served as discriminative stimulus. The lever (3 cm by 5 cm) was located beneath the light. Food pellets (Noyes Precision Pellets PJFSC-0045, Research diets) were delivered by a food dispenser placed outside the chamber. All operant chamber events were recorded by ABET II Operant Chamber software. To change the context in the test phase of the Sleep/DiffCont and S-Depriv/DiffCont groups, the floor, walls, and orientation of the chamber in the room were changed. The floor was covered with sandpaper, the walls were covered with a checkered pattern of 4 cm by 4 cm black/white squares, and the whole chamber was rotated by 90 degrees in counterclockwise manner.

### Data reduction and statistical analyses

Cumulative sleep time was determined offline from videotaped retention intervals using Camtasia Studio 8.0 video software (Techsmith, USA), with sleep identified whenever the rat displayed a typical sleep posture and stayed immobile for at least 5 s. Validity of this sleep scoring procedure was demonstrated in previous studies in rats and mice showing agreement of 92 % to standard EEG-/EMG-based scoring (e.g., Van Twyver et al. 1973; Pack et al. 2007). This high agreement concurs with our own observations in rats using both visual and EEG-/EMG-based scoring of sleep; typically, visual scoring slightly underestimates sleep time mainly because short-sleep bouts (<5 s) interrupted by movements can remain undetected. For the purpose of the present experiments, we considered visual sleep scoring optimal because we did not aim at differentiating specific sleep stages, and additional stress due to surgeries and electrode implants could be avoided.

To assess memory performance on the conditional discrimination task in the test phase, for each rat, the number of trials needed to achieve the learning criterion was determined. Additionally, for each rat, a discrimination ratio was calculated across all trials of the test phase as follows: Discrimination ratio = (mean number of responses during S1/sec − mean number of responses during S2/sec)/(mean number of responses during S1/sec + mean number of responses during S2/sec).

For statistical analyses, SPSS software was used (IBM, Armonk, NY, USA). Analyses basically relied on analyses of variance (ANOVA) which included group factors representing the brain state during the “retention condition” (sleep, sleep deprived, wake) and the “context” condition at the test phase (same vs different context). Because S-Depriv/SameCont and Wake/SameCont groups were closely comparable with regard to all target variables, data from these groups were collapsed, and the main analyses were then based on 2 × 2 ANOVA, i.e., two retention conditions (sleep vs sleep deprivation or wake) and 2 context conditions (same vs different). Separate analyses, including only the S-Depriv/SameCont group rather than the pooled S-Depriv/SameCont and Wake/SameCont groups, yielded essentially the same results and are not reported here. Analyses were performed on the number of trials needed to reach the learning criterion, the discrimination scores, and additionally on the number of responses during S1 and S2, separately. Significant ANOVA effects were followed by post hoc *t* tests. Regarding the discrimination ratios, one-sample *t* tests confirmed (*p* < 0.001) that for each group performance was above chance level. Finally, Pearson’s correlation coefficients were calculated between the sleep time and behavioral parameters during the test phase. A *p* value <0.05 was considered significant.

## Results

During the learning phase, performance on the go/nogo conditional discrimination task was closely comparable in all groups. All groups needed similar numbers of trials to achieve the learning criterion (Sleep/SameCont 31.36 ± 2.23; S-Depriv/SameCont 32.30 ± 1.51; Wake/SameCont 32.30 ± 2.24; Sleep/DiffCont 31.00 ± 2.21; and S-Depriv/DiffCont 29.30 ± 1.79; all *p* > 0.28). Also, discrimination ratios were comparable for the five groups (all *p* > 0.7).

Analyses of relearning of the conditional discrimination during the test phase revealed highly comparable performance for the S-Depriv/SameCont and Wake/SameCont groups (*p* > 0.35, for all comparisons) which led us to pool data from these groups and to analyze effects of the retention conditions and context conditions during the test phase in a 2 (sleep vs sleep deprivation) by 2 (same vs different context) ANOVA. Importantly, sleep compared with sleep deprivation or wakefulness during the retention interval significantly reduced the number of trials needed to achieve the learning criterion during the test phase [*F*(1, 47) = 32.78, *p* < 0.001, for main effect of retention condition, Fig. 1b]. The benefit from sleep was independent of the context during the test phase (*p* = 0.83, for retention × context interaction). However, tested in a different context, the animals generally needed more trials to reach the learning criterion than when tested in the same context as that during the learning phase [*F*(1, 47) = 20.80, *p* < 0.001, for main effect of context]. The beneficial effect of sleep on conditional discrimination memory was further confirmed by post hoc pairwise comparisons between groups, indicating that the Sleep/SameCont group required significantly less trials to achieve the learning criterion compared with all other groups (all *p* < 0.01, Fig. 1b). Also, the Sleep/DiffCont was significantly faster in achieving the criterion than the S-Depriv/DiffCont group (20.7 ± 1.3 vs. 27.8 ± 1.7 trials, respectively; *p* < 0.01). A quite similar pattern was obtained for the discrimination ratio during the test phase, where ANOVA also revealed strong main effects for both the retention condition [*F*(1, 47) = 23.60, *p* < 0.001] and the context condition [*F*(1, 47) = 46.67, *p* < 0.001]. Additionally, the retention × context interaction yielded significance [*F*(1, 47) = 4.68, *p* < 0.05], indicating that the sleep-induced enhancement in the discrimination ratio, with reference to sleep deprivation was slightly greater when tested in the same context than during testing in a different context. Accordingly, post hoc analyses revealed that discrimination ratios in the Sleep/SameCont and S-Depriv/SameCont groups differed significantly [*t*_(29)_ = 7.71, *p* < 0.001], whereas the corresponding difference between the Sleep/DiffCont and S-Depriv/DiffCont was not significant [*t*_(18)_ = 1.51, *p* = 0.15]. Furthermore, post hoc comparisons confirmed a higher discrimination ratio after sleep when testing was in the same context than when testing took place in a different context [Sleep/SameCont vs Sleep/DiffCont *t*_(19)_ = 8.90, *p* = 0.001].

**Fig. 2:**
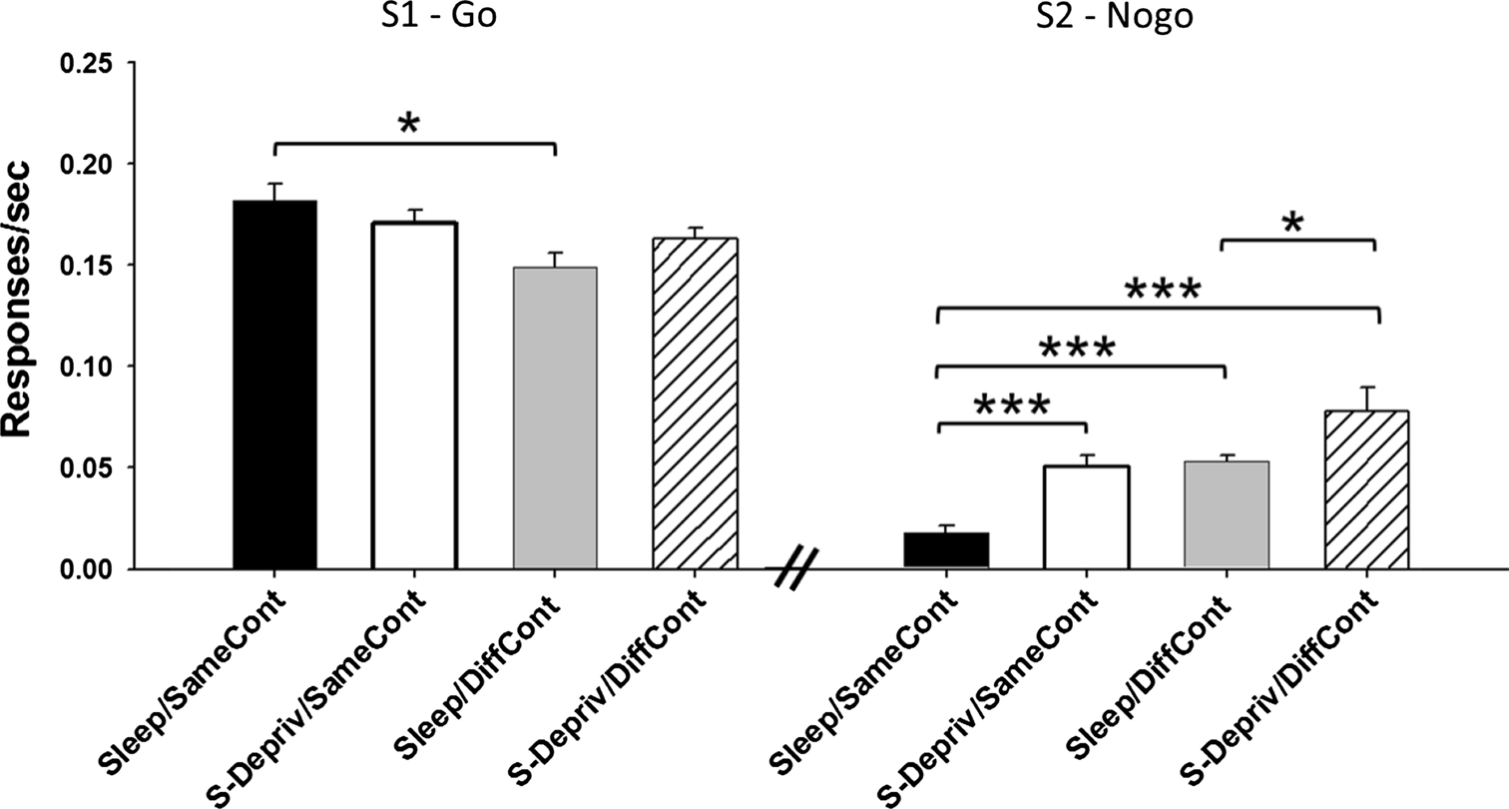
Mean (±SEM) responses (per second) during S1 (Go, *left panel*) and S2 (Nogo, *right panel*) of the conditional discrimination task at the test phase, separately for the Sleep/SameCont, the S-Depriv/SameCont, the Sleep/DiffCont, and the S-Depriv/DiffCont groups. An effect of sleep was revealed only for the response rate to S2 which improved (i.e., decreased) after sleep, with this effect being independent of the context in which the animal was tested. ***p < 0.001; **p < 0.01; *p < 0.05, for pairwise comparisons between groups

A more fine-grained analysis performed separately on the number of responses during S1 (i.e., the Go stimulus) and S2 (Nogo) revealed that the profit from sleep for conditional discrimination memory primarily originated from an improvement in the Nogo responses to S2 (Fig. 2). For S1, the number of (correct) responses was increased when the test phase was conducted in the same context as during learning compared to testing in a different context [*F*(1, 47) = 7.29, *p* = 0.01, for the main effect of context]. However, there was no significant main effect for the retention condition (*p* = 0.80), and also, the retention × context interaction failed to reach significance [*F*(1, 47) = 2.83, *p* = 0.1]. The pattern was confirmed by post hoc pairwise tests which only revealed a significant difference between the Sleep/SameCont and the Sleep/DiffCont groups [*t*_(19)_ = 2.90, *p* < 0.01]. By contrast, analyses of the (false) responses to S2 (Nogo) revealed a strong beneficial reducing effect on such responses, after sleep [*F*(1, 47) = 20.67, *p* < 0.001, for main effect of retention], in addition to a main effect of context [*F*(1, 47) = 22.34, *p* = 0.001]. Importantly, the retention × context interaction was not significant (*p* = 0.61), indicating that the improvement in response inhibition produced by sleep was independent of the context of testing (same or different from that during learning). Indeed, average decreases in responses after sleep (with reference to sleep deprivation) were fairly comparable for testing in the same context versus different context as during learning (0.037 vs 0.026 responses/sec). Post hoc pairwise tests confirmed significantly lower response rates to S2 in the Sleep/SameCont than in the S-Depriv/SameCont [*t*_(29)_ = −4.63, *p* < 0.001], as well as in the Sleep/DiffCont compared with the S-Depriv/DiffCont group [*t*_(18)_ = −2.19, *p* < 0.05]. Moreover, after sleep retention intervals, testing in the same context produced less false responses to S2 than testing in a different context [*t*_(19)_ = −7.87, *p* < 0.001].

Sleep during the retention intervals did not differ between the Sleep/SameCont and Sleep/DiffCont groups (sleep onset 35.21 ± 4.80 vs 39.11 ± 4.04 min; *p* = 0.54; sleep duration 25.63 ± 2.75 vs 19.53 ± 1.94 min, *p* = 0.09). There were also no significant correlations between sleep onset or sleep time and any of the behavioral parameters [*r* < 0.29, *p* > 0.14].

## Discussion

Our data indicate an enhancing effect of sleep after training on memories for nogo/go responses in a conditional discrimination task. In the relearning test, rats needed fewer trails to reach the learning criterion and showed overall a better discrimination of go and nogo responses when they had slept during the 80-min retention interval after training then when they had stayed awake during this interval. Sleep mainly benefitted nogo response learning, while effects of sleep on the learning of go responses per se were not significant. When the animal was tested in the same context as during learning, relearning was generally enhanced as compared to testing in a different context. However, effects of sleep on the main dependent variable did not interact with those of context, i.e., sleep improved memory for discriminative nogo responses independent of the original context in which the training took place. These results add to the notion that sleep essentially contributes to the consolidation of memory that during encoding involves prefrontal–hippocampal circuitry, i.e., the presumed neuroanatomical basis of episodic encoding (Battaglia et al. 2011; Inostroza and Born 2013). They demonstrate behavioral inhibition learning as a feasible rat model for the study of sleep-dependent system consolidation.

Because prefrontal executive control functions like behavioral inhibition have been considered particularly vulnerable to sleep deficits (e.g., Durmer and Dinges 2005, but see Bratzke et al. 2012), it could be argued that relatively weaker task memory after sleep deprivation was a consequence of the increased time the rats had to spent wake before testing and of increased fatigue. However, we tested a separate group of rats on an 80-min retention interval that took place in the evening hours, i.e., the rats’ active phase when they were spontaneously awake during the retention period. These rats presumably not suffering from any fatigue showed memory performance at testing entirely indistinguishable from the rats of the sleep deprivation group, which were tested after 80-min retention periods in the morning hours and in which wakefulness was enforced by gentle handling. Thus, the group of rats tested in the active phase argues against a purely fatigue-mediated response-inhibition deficit. Nevertheless, it could be argued that sleep might nonspecifically strengthen response inhibition. Yet, such nonspecific effect would be expected to result in a generally reduced response rate across both go and nogo stimuli, rather than in a specific response reduction to the nogo stimulus as observed here. This outcome in combination with the fact that not responding to the nogo stimulus was not reinforced in our task speaks in favor of the view that sleep benefitted the memory for a specific inhibitory response rather than acted by improving capabilities to inhibit motor behavior in general.

Memory performance at test being highly comparable between the wake and sleep deprivation groups does not only exclude fatigue as a confound of performance in the sleep deprivation groups, but also possible confounding influences on memory performance conveyed by the circadian rhythm or the stress of sleep deprivation. The latter confound is all the more unlikely in light of the gentle handling procedure which we used for sleep deprivation and which typically prevents any substantial increases in the release of stress hormones if applied during relatively short intervals of less than 2 h (Hagewoud et al. 2010). From a different perspective, it could be argued that the 80-min retention interval used in our study was too short to allow for long-term consolidation processes to be fully expressed. We established the 80-min duration of the retention interval in previous experiments which demonstrated robust effects of intervals of this length filled with sleep versus wakefulness on the retrieval of episodic-like memories in rats (Inostroza et al. 2013). Although with this relatively short retention interval testing focused on the consolidation of intermediate-term memory (Kesner and Hunsaker 2010), that study also provided hints that findings could be generalized to long-term consolidation, as the enhancing effects of sleep on episodic memory did not change when retesting took place after an extended delay of about 3 h. There is currently no evidence arguing against extrapolating sleep-induced memory benefits obtained after retention intervals of intermediate length to the long-term retention of respective behaviors. Nevertheless, as the underlying plastic neuronal processes probably differ, the present effects of sleep on conditional discrimination learning need to be confirmed in studies using distinctly longer retention intervals. Such studies might also test whether additional intervals (windows) exist at longer delays where inhibitory memory becomes sensitive to the disrupting effects of sleep deprivation (Smith 1995; Fogel et al. 2009).

The present data of robust sleep-dependent improvements for conditional discrimination memories add to a rapidly growing body of research indicating a consolidating effect of sleep specifically for memories that are encoded through the prefrontal–hippocampal system required for the acquisition of explicit and episodic-like memories. Beneficial effects of sleep on these memories have so far been more thoroughly studied in humans than rodents (e.g., Robertson et al. 2004; Wilhelm et al. 2011; Inostroza et al. 2013; Rasch and Born 2013), although rodents are commonly used to clarify the underlying mechanism of the consolidation process during sleep (e.g., Wilson and McNaughton 1994; Benchenane et al. 2010). Against this backdrop, the present study proves that response-inhibition learning might be a promising behavioral model for the study of the mechanisms that mediate the putative system consolidation process hippocampal memories undergo during sleep.

The employed go/nogo conditional discrimination task of this study is particular as it is not an episodic memory task in the strict sense, but, nevertheless, in involving a behavioral response, inhibition feature essentially relies on prefrontal–hippocampal circuitry (Chudasama et al. 2012). It is likely that prefrontal–hippocampal networks contributing to the encoding of response inhibition during learning reactivate during slow wave sleep after learning (Benchenane et al. 2010; Peyrache et al. 2009). Effects of sleep on learned response inhibition have so far been mainly examined using fear extinction paradigms. The studies consistently showed a beneficial effect of sleep on fear extinction learning in rats and humans that was linked to both signs of NonREM sleep and REM sleep (e.g., Pace-Schott et al. 2012; Spoormaker et al. 2012; Kleim et al. 2013; Datta and O’Malley 2013; Fu et al. 2007; Silvestri 2005). Whereas the present study is limited in that it did not apply electrophysiological recordings to differentiate specific sleep stages, it adds to those previous observations in demonstrating a robust sleep benefit for learned response inhibition using an appetitive, rather than aversive, behavioral approach in which inhibitory memory might be differently mediated. Indeed, our data reveal that it is the nogo component of the conditional discrimination task which is critically enhanced by the sleep-associated consolidation process, with effects of sleep on the go component remaining negligible. We speculate that the involvement of such components of prefrontal executive control in a task makes respective memories particularly susceptible for entering sleep-dependent consolidation (Wilhelm et al. 2011; Diekelmann et al. 2013; Born and Wilhelm 2012), although the plastic influence of sleep on executive function per se is presently not well understood (Kuriyama et al. 2008).

An important finding of the present study is that the benefitting effect of sleep on conditional discrimination memory was generalized to testing in a context different from that during training. Note a prerequisite for this generalization is that the different context per se is familiar to the rat, because in pilot studies, we found that using a novel context at testing completely suppressed any expression of discrimination memories, presumably due to predominant exploratory behavior. Sleep facilitating generalization of learned inhibitory behaviors has been likewise demonstrated for conditioned fear extinction in humans (Pace-Schott et al. 2009, 2012). Also, in humans, Deliens et al. (2013) revealed an enhancing effect sleep on word memories when recall was tested in a different emotional context, i.e., after experimentally inducing a mood different from that during encoding, and Cairney et al. (2011) reported signs of an even superior memory effect of sleep (with reference to wakefulness) when recall for words was tested in a different context than in the same environmental context as during learning. Against this background of human studies, the present study appears to be the first to demonstrate in a rat model, that sleep induces a robust enhancement of memory that is independent from the learning context. Indeed, this pattern was remarkably clear for memory of the inhibitory nogo component where sleep-induced gains in memory (with reference to sleep deprivation) were comparable for testing in the different and same contexts, whereas discrimination ratios revealed a greater sleep benefit for recall in the same context. In fact, this pattern well agrees with the concept of an active system consolidation process during sleep which is triggered by repeated memory reactivations spreading from hippocampal to extra-hippocampal networks and serves two different functions (Inostroza and Born 2013), to enhance the context bound (episodic) representation of memory within hippocampal circuitry as well as to enhance the memory for an event per se, independent of its context. The latter function might help unbind the memory from its context and promote the formation of de-contextualized schema-like memories residing in extra-hippocampal networks.
